# The Effect of Catgut Embedding at Acupoints Versus Nonacupoints in Abdominal Obesity: Protocol for a Multicenter, Double-Blind, 16-Week Randomized Controlled Trial

**DOI:** 10.2196/46863

**Published:** 2023-07-10

**Authors:** Qifu Li, Gaoyangzi Huang, Xianmei Pei, Xin Tang, Renrui Zhang, Ya Huang, Zili Liu, Rong Yi, Chonghui Xing, Xinghe Zhang, Taipin Guo

**Affiliations:** 1 School of Second Clinical Medicine The Second Affiliated Hospital Yunnan University of Chinese Medicine Kunming China; 2 Department of Acupuncture and Moxibustion Kunming Hospital of Traditional Chinese Medicine Kunming China; 3 Department of Acupuncture and Moxibustion The Sports Trauma Specialist Hospital of Yunnan Province Kunming China

**Keywords:** acupoint catgut embedding, nonacupoints, abdominal obesity, randomized controlled trial, protocol, waist circumference

## Abstract

**Background:**

Obesity is an increasing problem worldwide. The effective treatments for obesity mainly include diet, physical activity, behavioral intervention, pharmacotherapy, and bariatric surgery, which all have certain limitations. As a specific type of acupuncture therapy, acupoint catgut embedding (ACE) has gained substantial attention in the management of obesity in recent years. Previous studies suggested that ACE may be an effective obesity treatment. However, the evidence for the efficacy of ACE in abdominal obesity (AO) remains inadequate due to the paucity of high-quality studies.

**Objective:**

This study aims to investigate the difference in the effectiveness of catgut embedding at acupoints and catgut embedding at nonacupoints in patients with AO and to further validate the efficacy and safety of ACE for AO.

**Methods:**

This is a multicenter, double-blind, 16-week randomized controlled trial. A total of 92 eligible participants with AO will be randomly divided into 2 groups (1:1 allocation ratio). The ACE group will receive catgut embedding at acupoints and the control group will receive catgut embedding at nonacupoints. The intervention will be performed every 2 weeks for a total of 6 sessions. Follow-up will be performed every 2 weeks for a total of 2 visits. The primary outcome is waist circumference. Secondary outcomes include body weight, BMI, hip circumference, and the visual analog scale of appetite. Upon the completion of the trial, we will evaluate the effect of catgut embedding at acupoints or nonacupoints on obesity indicators in patients with AO. For treatment outcomes, an intention-to-treat analysis will be performed.

**Results:**

The start of recruitment began in August 2019 and is expected to end in September 2023.

**Conclusions:**

Although studies have been conducted to demonstrate the effectiveness of ACE in the treatment of obesity, the evidence for the efficacy of ACE in AO remains insufficient due to the quality of the studies. This rigorous normative randomized controlled trial will verify the effect of catgut embedding at acupoints or nonacupoints in patients with AO. The findings will provide credible evidence as to whether ACE is an effective and safe treatment for AO.

**Trial Registration:**

Chinese Clinical Trial Registry ChiCTR1800016947; https://tinyurl.com/2p82257p

**International Registered Report Identifier (IRRID):**

DERR1-10.2196/46863

## Introduction

Obesity has become a global health issue regardless of race, region, and age [1]. Globally, the prevalence of obesity in adults was approximately 13% (603.7 million). There were approximately 107.7 million children and adolescents who were obese [2,3]. The prevalence of obesity was about 20% or more in the United States, the Middle East and North Africa, the Caribbean, Polynesia, and Micronesia [4]. The increase of adults who are obese has leveled off in several high-income countries, but the incidence is dramatically on the rise in most low- and middle-income countries, particularly in urban regions [1,5]. As a major type of obesity, the prevalence of abdominal obesity (AO) in adults was 29.1% (28.6% in men and 29.6% in women) in China [6]. Obesity is a worldwide problem and a burden in many diseases [2,7,8]. Studies have demonstrated that obesity may lead to diabetes, subfertility, chronic spontaneous urticaria, nonalcoholic fatty liver disease, Alzheimer disease, cardiovascular diseases, and even cancer [2,9-18]. In addition to diseases, obesity can also affect disability-adjustment life-years and generate a large economic burden [4,19-21]. Obesity is also a persistent problem, where most children who are obese will remain obese in adolescence [22].

The effective treatments for obesity mainly include diet, physical activity, behavioral intervention, pharmacotherapy, and bariatric surgery [23-29]. Diet and physical activity require great self-discipline and enough time, which are difficult to adhere to. The effect of behavioral intervention for obesity is typically limited [30]. Side effects such as abdominal pain, vomiting, nervousness, insomnia, headache, dizziness, dysgeusia, and insomnia may accompany pharmacotherapy [31]. Pharmacotherapy could not be used for patients who are obese with complicated active cardiovascular disease, glaucoma, uncontrolled hypertension, anorexia, thyroid, and many other diseases [32,33]. For pregnant individuals and children, the safety of pharmacotherapy is still not guaranteed currently [34]. Bariatric surgery requires a high level of medical technology and may increase the risk of fractures [35-37]. Alternative therapies such as acupuncture, moxibustion, acupoint catgut embedding (ACE), and massage are increasingly used for the treatment of obesity [38-42].

ACE is a special acupuncture therapy that combined the absorbable surgical suture and acupoint, and the effective stimulation of every ACE treatment could persist for 2 weeks. Previous studies indicated that ACE was an efficient treatment for obesity, but the risk of bias was significantly high [43]. Therefore, this rigorous and normative randomized controlled trial (RCT) study is designed to verify the efficiency of catgut embedding at acupoints and nonacupoints in participants with AO. The findings will provide credible evidence as to whether ACE is an effective and safe treatment for AO.

## Methods

### Study Design and Setting

**Figure 1:**
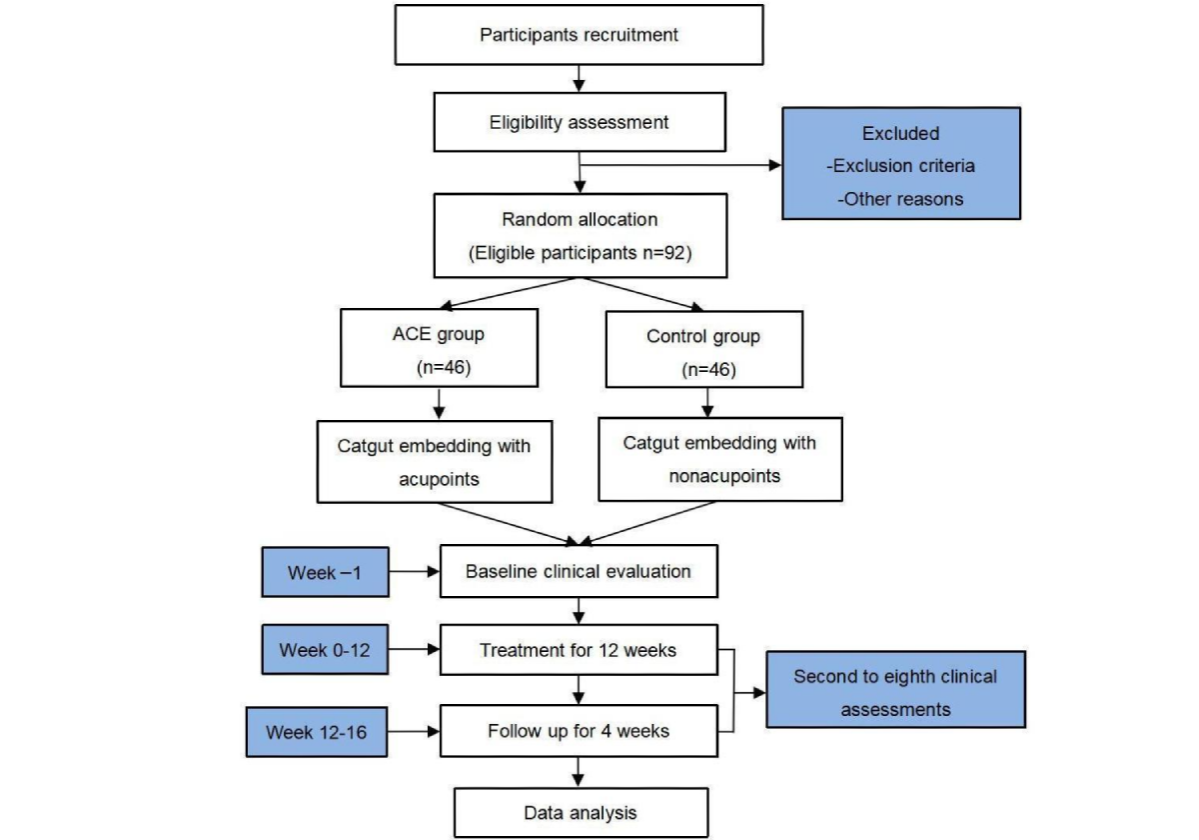
Flowchart of the trial. ACE: acupoint catgut embedding.

This multicenter, double-blind, 16-week RCT will be conducted from August 2019 to September 2023 in 4 clinical centers: The Second Affiliated Hospital of Yunnan University of Chinese Medicine, The Sports Trauma Specialist Hospital of Yunnan Province, Kunming Hospital of Traditional Chinese Medicine, and ShengAi Hospital of Traditional Chinese Medicine. A total of 92 eligible participants with AO will be assigned to 1 of 2 groups (catgut embedding at acupoints or nonacupoints) using central randomization (1:1 allocation ratio). Every participant will receive a 12-week intervention period and a 4-week follow-up. The flowchart of this study is shown in Figure 1.

### Ethics Approval

This protocol is in line with the Declaration of Helsinki and has been approved by the Hospital Ethics Committee of The Sports Trauma Specialist Hospital of Yunnan Province (2018CK-001) in June 2018. The study has been registered in the Chinese Clinical Trial Registry (ChiCTR1800016947). This protocol is compliant with the principles of the SPIRIT (Standard Protocol Items: Recommendations for Interventional Trials; see Multimedia Appendix 1) and CONSORT (Consolidated Standards for Reporting Trials) guidelines [44]. The SPIRIT checklist is presented in Multimedia Appendix 1 and the schedule is shown in Table 1.

**Table 1:** Study schedule for data measurements.

			Study period and time point (week)										
		Enrollment		Allocation	Treatment phase							Follow-up	
		–1		0	2	4	6	8	10	12	14		16
**Enrollment**													
	Eligibility screen	✓											
	Informed consent	✓											
	Physical examination	✓											
	Randomization			✓									
**Interventions**													
	ACE^a^ group (n=46)				✓	✓	✓	✓	✓	✓	✓		✓
	Control group (n=46)				✓	✓	✓	✓	✓	✓	✓		✓
**Assessments**													
	BW^b^	✓		✓	✓	✓	✓	✓	✓	✓	✓		✓
	BMI	✓		✓	✓	✓	✓	✓	✓	✓	✓		✓
	WC^c^	✓		✓	✓	✓	✓	✓	✓	✓	✓		✓
	HC^d^			✓	✓	✓	✓	✓	✓	✓	✓		✓
	VAS^e^ of appetite			✓	✓	✓	✓	✓	✓	✓	✓		✓
**Safety**													
	AE^f^				✓	✓	✓	✓	✓	✓			

^a^ACE: acupoint catgut embedding.

^b^BW: body weight.

^c^WC: waist circumference.

^d^HC: hip circumference.

^e^VAS: visual analogue scale.

^f^AE: adverse event.

### Participants

Participants who meet all the following criteria will be included: (1) aged between 18 and 60 years with simple obesity; (2) BMI ≥28 kg/m^2^ and waist circumference (WC) ≥90 cm for male participants and ≥85 cm for female participants; (3) agreed to participate in this study and signed written informed consent for this trial and catgut embedding therapy; and (4) did not participate in other trials within past 3 months. Participants will be excluded if they meet any one of the following criteria: (1) secondary obesity, such as obesity caused by endocrine disease (Cushing syndrome, thyroid disease, hypothalamic disease, pituitary disease, gonadal disease, etc) and medication (glucocorticoid or antipsychotics); (2) pregnancy, lactation, and childbirth within the past 6 months; (3) heart disease; hematopoietic system, liver, kidney, and other important organ diseases; and hypertension that is not effectively controlled; (4) severe mental and neurological diseases where the patient is unable or unwilling to cooperate; (5) allergy to alcohol and animal protein or immune diseases; and (6) received other weight loss treatment within the past 3 months.

### Criteria for Elimination From the Study

Those who meet the following criteria should be eliminated from the study: (1) misdiagnosis and misrepresentation; (2) poor treatment tendency; (3) involvement with other treatments after being selected; and (4) participants’ withdrawal before the first test recording.

### Recruitment and Randomization

All participants with eligible AO will be recruited from the 4 hospitals and the community. Recruitment methods mainly include posters, public notice boards, and WeChat public numbers in hospitals and communities. The randomization sequence will be generated by the Clinical Research Center of Yunnan University of Chinese Medicine using a computerized random number generator and placed in opaque envelopes. Stratified randomization will be performed for the 4 clinical centers. To ensure allocation concealment, randomization will be performed by an independent researcher. The research assistant will open the envelope when the participant begins treatment to determine the assigned group and will not disclose grouping information under any circumstances. The acupuncture therapist will be responsible for manipulation only.

### Blinding and Informed Consent

Participants will be informed that they have a 1 in 2 chance of being assigned to 1 of 2 treatments: catgut embedding at acupoints or nonacupoints. The procedure and stimulation of the catgut embedding at acupoints are identical to catgut embedding at nonacupoints. Therefore, participants will be blinded to their treatment assignment. Both groups of participants will be assigned to different treatment rooms to prevent intercommunication. In addition, the acupuncturist, outcome assessor, and those involved in data collection and statistics will be blinded to treatment assignment throughout the trial. Research assistants with knowledge of grouping information will be asked not to communicate with participants or outcome assessors regarding treatment procedures and responses. All patients will be fully informed about the study (including the purpose of the trial and possible risks, etc) prior to participation in the trial, and they have the right to withdraw from the trial at any time without giving a reason. They will sign a written informed consent for voluntary participation prior to enrollment.

### Sample Size Calculation

According to the existing literature [45], the mean BMI of simple obesity was 32.30 kg/m^2^, whereas after 12 weeks and 6 treatment of acupuncture combined with a low-energy diet, the mean BMI was 30.98 kg/m^2^, and the improvement value was 1.32 kg/m^2^. In contrast, the improvement value was 1.02 kg/m^2^ in a control group with a variation from 32.74 kg/m^2^ to 31.73 kg/m^2^ when treated by sham acupuncture combined with a low-energy diet, and the SD of the 2 groups was 0.31 kg/m^2^. In this project, the expected improvement value of the mean BMI of the Shu-Mu acupoints embedding group after treatment is 1.14 kg/m^2^ and that of the nonacupoints embedding group is 0.9 kg/m^2^, with the SD of each group being 0.31 kg/m^2^. The significant level is ɑ=.05, and the power of a test is 1 – β = 0.95. The sample size is 84 as calculated by German G*power software 3.1 [46]. According to the 10% dropout, the total sample is 92 cases, and 46 cases will be assigned to each group.

### Interventions

All catgut embedding manipulations will be performed by an acupuncturist with national medical qualifications. The participants will receive treatment every 2 weeks for a total of 6 sessions.

### ACE Group

**Figure 2:**
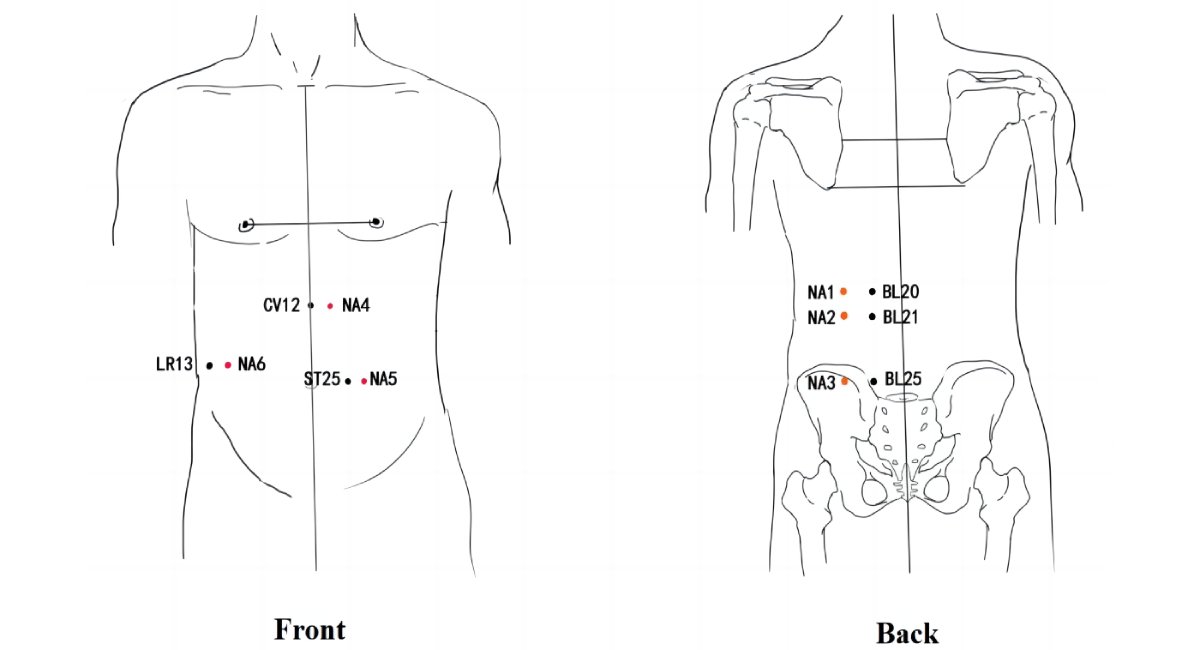
Locations of acupoints and nonacupoints (NAs). Acupoints: BL20: Pishu, BL21: Weishu, BL25: Dachangshu, CV12: Zhongwan, LR13: Zhangmen, and ST25: Tianshu.

The selection of acupoints for treatment in the ACE group will be based on the theory that obesity is closely related to the spleen, stomach, and large intestine in traditional Chinese medicine and the Shu-Mu acupoints allocation method commonly used in the current clinical treatment of obesity. The acupoint prescription includes BL20 (Pishu), BL21 (Weishu), BL25 (Dachangshu), RN12 (Zhongwan), ST25 (Tianshu), and LR13 (Zhangmen). All acupoints except Zhongwan are selected bilaterally, for a total of 11 acupoints. The locations of these acupoints are shown in Table 2 and Figure 2.

**Table 2:** The locations of acupoints.

Acupoints	Location
BL20 (Pishu)	In the upper back region, at the same level as the inferior border of the spinous process of the 11th thoracic vertebra (T11), 1.5 B-cun lateral to the posterior median line
BL21 (Weishu)	In the upper back region, at the same level as the inferior border of the spinous process of the 12th thoracic vertebra (T12), 1.5 B-cun lateral to the posterior median line
BL25 (Dachangshu)	In the lumbar region, at the same level as the inferior border of the spinous process of the fourth lumbar vertebra (L4), 1.5 B-cun lateral to the posterior median line
CV12 (Zhongwan)	On the upper abdomen, 4 B-cun superior to the center of the umbilicus, on the anterior median line
ST25 (Tianshu)	On the upper abdomen, 2 B-cun lateral to the center of the umbilicus
LR13 (Zhangmen)	On the lateral abdomen, inferior to the free extremity of the 11th rib

### Control Group

The acupoints in the control group will be selected based on the acupoints in the ACE group by moving each acupoint 2-3 cm outward horizontally in the median line (CV12 to the left side only) as a nonacupoints (not located on or overlapping with other meridians). They are labeled NA1, NA2, NA3, NA4, NA5, and NA6, respectively, for a total of 11 acupoints. The locations of these acupoints are shown in Table 3 and Figure 2, marked as red points.

**Table 3:** The location of nonacupoints (NAs).

Nonacupoint	Location
NA1	The sitting position of the patient, in alignment with BL20 and the midpoint of the first and second lateral line of the bladder channel
NA2	The sitting position of the patient, in alignment with BL21 and the midpoint of the first and second lateral line of the bladder channel
NA3	The sitting position of the patient, in alignment with BL25 and the midpoint of the first and second lateral line of the bladder channel
NA4	The supine position of the patient, in alignment with RN12 and the midpoint of the left kidney and stomach channel
NA5	The supine position of the patient, in alignment with ST25 and the midpoint of the stomach and spleen channel
NA6	The sitting position of the patient, in alignment with LR13; a vertical line is made from Zhangmen to the spleen channel and the midpoint of the vertical line

### Operation Instruments

The thread-embedding needle used in this study is an 8# disposable needle (Jiangxi Glance Medical Equipment Co Ltd Production), and the medical protein string is an absorbable collagen line with the specification of 2-0, 2 cm × 20 length (Jiangxi Longteng Biotechnology Co, Ltd).

### Manipulation

The specific manipulation is as follows: participants are instructed to be placed in an appropriate position; the separate research assistant marks the acupoints or nonacupoints and routinely disinfects the skin around them; and a sterile sheet is spread, leaving only the buried area. Then, acupuncturists, who are unaware of the group allocation, take a sterile protein cord 1-2 cm long (the length depends on the location of the acupoints), place it on the front end of the trocar, connect the needle core, lift the partial skin with the thumb and forefinger of one hand, and pierce the needle with the other hand. When the piercing reaches the desired depth, apply appropriate push-and-twist techniques, push the needle core and remove the needle tube, and implant the sterile protein string in the subcutaneous tissue or muscle layer of the acupoints. After removing the needle, press the needle hole with a dry cotton ball for half a minute to stop the bleeding. Meanwhile, check that there is no thread residue exposure and no bleeding and then paste a bandage to protect the needle hole. It will be recommended that patients do not take a bath for 24 hours and keep the embedding place dry.

### Outcomes and Assessment

#### Primary Outcome

The primary outcome is the change of WC every 2 weeks, from baseline to end points. To collect the measurements, participants will stand their body upright, feet at shoulder width. An inelastic, 1-mm minimal tapeline will be used, set on the midpoint between the upper border hipbone and lower border 12th rib of the right midaxillary line. At the end of normal exhalation, they will be measured around the abdomen in the horizontal direction and without pressing the skin. The WC will be measured in millimeters (mm) and accurate to 1 mm.

#### Secondary Outcomes

**Figure 3:**
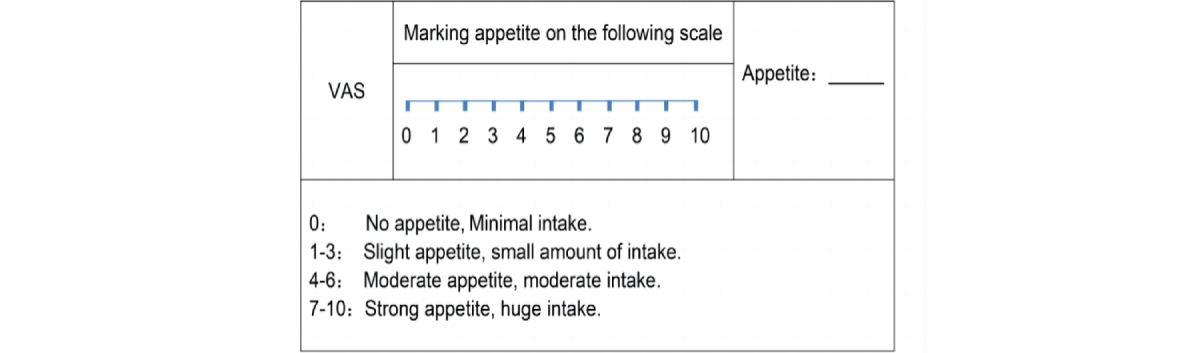
The visual analogue scale (VAS) of appetite.

The following secondary outcomes will be assessed:

1. The change of body weight (BW) will be measured every 2 weeks, from baseline to end points. Participants will be fasting 2 hours before the assessment. Shoes will be taken off, and only light clothing should be worn in a warm room while standing upright in the middle of the calibrated scale. BW will be measured in kilograms (kg) and accurate to 10 g.
2. The change of BMI will be measured every 2 weeks, from baseline to end points: *BMI = BW (kg) ÷ height2 (m2)*. Height will be measured after the weight measurement, with the participants standing upright and close to the measuring stick. Height will be measured from the top of the head to the bottom of the feet in meters (m) and accurate to 1 mm.
3. The change of hip circumference (HC) will be measured every 2 weeks, from baseline to end points. HC is the circumference of the most prominent point of the pelvis. Participants will stand upright, feet at shoulder width. The circumference at the end of the normal exhalation will be measured in millimeters (mm) and accurate to 1 mm.
4. The visual analog scale (VAS) of appetite will be scored every 2 weeks, from baseline to end points. Appetite will be assessed by VAS as reported in Doucet et al [47] (see Figure 3): no appetite and minimal intake (score of 0), slight appetite and a small amount of intake (score of 1-3), moderate appetite and moderate intake (score of 4-6), and strong appetite and huge intake (7-10 score of 7-10).

All of the above measurements and evaluations will be performed by a separate researcher in the morning, with the patient coming to the hospital without eating breakfast.

#### Safety Evaluation

Due to puncture injury and protein wire stimulation, participants may experience local sterile inflammatory reactions such as redness, swelling, fever, pain, or small amounts of exudate within 1-5 days, which are normal, generally do not require treatment, and are kept under close observation. If there is more exudate from the acupoints; localized redness; swelling; increased pain; increased temperature; or even protein line spillage, wound fat liquefaction, or nerve damage, treatment will be suspended when these adverse reactions occur. Participants will be treated aggressively until they have fully recovered and reported to the study center for evaluation by a medical professional to see if the trial can continue. Serious or unexpected adverse events will be verbally reported within 24 to 48 hours. All information will be classified as a safety event.

#### Data Management and Quality Control

The outcome evaluator will complete the initial data in the case report form and enter it into a Microsoft Excel spreadsheet. The protocol will be reviewed several times by an acupuncturist with the title of director and a statistical expert. To ensure the smooth conduct of the study, the study leader shall provide uniform training to all study personnel before the start of the study and provide detailed operational training on the project implementation protocol and observation indicators to familiarize them with the study process and specific implementation details to ensure the reliability of the study results. The project leader shall supervise and check the entire study process to confirm that all study data and case report forms are recorded and reported truthfully, accurately, completely, and consistently with the original data. Any deviation from the protocol will be reported to the ethics committee of The Sports Trauma Specialist Hospital of Yunnan Province, which will ultimately decide whether we must change the protocol or terminate the trial. To ensure participant compliance, all treatments and tests will be provided free of charge.

### Statistical Analysis

All data will be analyzed by SPSS statistical software (version 19.0; IBM Corp). The clinical outcomes and baseline characteristics are based on the intention-to-treat population, which will include participants who have received at least one treatment. Continuous variables will be shown as the mean and SD, whereas the nonnormally distributed variables will be presented as the median and IQR. Categorical variables will be described as numbers and percentages. The normality of continuous variables will be determined using the Shapiro-Wilk test, Kolmogorov-Smirnov test, skewness, and kurtosis. The homogeneity of continuous variables will be determined by *F*-test, Brown-Forsythe, and Bartlett tests. Comparisons between groups will be made using independent samples 2-tailed *t* tests or nonparametric tests (Mann-Whitney *U* test). Repeated measures within groups (≥3) will be analyzed using one-way ANOVA or nonparametric (Friedman) tests. Paired 2-tailed *t* tests or nonparametric tests (Wilcoxon test) will be used for within-group pre-post comparisons. For categorical data, the chi-square test will be used to assess the significance of differences. Values of *P*<.05 will be considered significant using a 2-sided test.

## Results

The start of recruitment began in August 2019 and is expected to end in September 2023.

## Discussion

### Expected Findings

In the past, obesity was considered a problem in high-income countries. However, recent studies reported that the prevalence of obesity is rapidly increasing in low- and middle-income countries [4,48-50]. As a type of obesity, AO is also related to cardiovascular diseases, cancer, and many other diseases [51-54].

ACE is a traditional Chinese medical practice that has been used in clinical practice since the mid-1960s. Compared with other treatments, ACE has the advantages of a short single-treatment time, long effective stimulation time, low impact on daily life, as well as being time-saving and low cost and having few side effects, which are well suited for the fast-paced modern society and become gradually favorable to patients who are obese. As a specific type of acupuncture therapy, ACE has gained substantial attention in the management of obesity in recent years. Research has demonstrated that ACE has a certain impact on obesity. First, it can reduce BW by regulating appetite and food intake. ACE stimulates specific acupoints, promoting the release of neurotransmitters and hormones to regulate feelings of hunger and satiety and ultimately reducing food intake [55]. Second, ACE can improve metabolic function. Studies have shown that acupoint stimulation can affect various physiological processes, including the endocrine, nervous, and immune systems, thus regulating energy and fat metabolism, improving insulin sensitivity, and enhancing blood lipid levels [41]. Additionally, ACE may also have positive effects on obesity by improving psychological well-being and sleep quality, among other factors [56]. However, current research has some limitations, including small sample sizes and inconsistent study designs [57]. Therefore, to better assess the effectiveness and safety of ACE treatment, further high-quality studies with larger sample sizes are needed.

The decrease of several indicators such as BW, WC, BMI, HC, and appetite is not only beneficial to personal health but also helps people who are obese to reshape their bodies [58]. Among them, BMI is the most commonly used indicator in obesity research, but it mainly reflects total body fat and cannot distinguish between fatty obesity and muscular obesity. HC is affected by both sex and age, and changes in abdominal fat cannot be accurately reflected promptly [59]. WC can directly reflect abdominal fat, so we use WC as the main outcome indicator and other related indicators as secondary outcome indicators. According to the classification adopted by the Chinese Medical Association of China in 2019, the inclusion criteria was set as WC ≥90 cm for male individuals and ≥85 cm for female individuals, rather than those of other Asian countries (WC ≥102 cm for male individuals and WC ≥88 cm for female individuals or WC ≥ 90cm for both male and female individuals) [6,60].

The combination of Shu and Mu acupoints is one of the classical methods of acupuncture and is mainly used in clinical practice to treat metabolic diseases of the viscera. According to the identification theory of traditional Chinese medicine, obesity is mainly caused by the dysfunction of the spleen, stomach, and large intestine, so we chose the Shu and Mu acupoints of these 3 internal organs as the treatment prescription. Our preliminary analysis of 175 obesity ACEs in the early stage also found that the existing high-frequency acupoints for obesity ACEs were mainly Shu and Mu acupoints of the spleen, stomach, and large intestine [61].

### Strengths and Limitations

First, this study is a multicenter RCT designed to investigate the efficacy and safety of ACE in AO with a relatively long study period, and the results will provide powerful evidence for the clinical application of ACE in the treatment of obesity. Second, participants’ diet and exercise will not be intervened, which will allow participants to maintain their original lifestyle habits throughout and maximize the efficacy of ACE. Finally, to maintain the success of the double-blind method, contact between participants from different groups will be avoided. The acupuncturist, data evaluator, and statistician will also be hidden from the group assignment, which will minimize possible bias in the results.

Limitations of this study are as follows. First, the relatively short follow-up period of this study may not be comprehensive enough to assess the durability of ACE efficacy. Second, the selection of nonacupuncture acupoints as an intervention in the control group in this study may inevitably produce some efficacy as well [62,63]. Third, only patients with AO are selected as the study population in this study, and further studies on different types of obesity will be conducted at a later stage.

### Conclusions

In summary, our trial aims to investigate the difference in the effectiveness of catgut embedding at acupoints and catgut embedding at nonacupoints in patients with AO. This finding will provide reliable evidence to support whether ACE is an effective and safe treatment for AO.

## Appendix

Multimedia Appendix 1 SPIRIT (Standard Protocol Items: Recommendations for Interventional Trials) checklist.

## Abbreviations

ACE: acupoint catgut embedding
AO: abdominal obesity
BW: body weight
CONSORT: Consolidated Standards for Reporting Trials
HC: hip circumference
RCT: randomized controlled trial
SPIRIT: Standard Protocol Items: Recommendations for Interventional Trials
VAS: visual analogue scale
WC: waist circumference

## Data Availability

The data sets generated or analyzed in this study will not be publicly available. Consent and ethical approval for this study do not include a provision for the sharing of data from this study.
